# Patients’ expectations of private osteopathic care in the UK: a national survey of patients

**DOI:** 10.1186/1472-6882-13-122

**Published:** 2013-05-31

**Authors:** CM Janine Leach, Anne Mandy, Matthew Hankins, Laura M Bottomley, Vinette Cross, Carol A Fawkes, Adam Fiske, Ann P Moore

**Affiliations:** 1 Clinical Research centre for Health Professions, University of Brighton, Aldro Building, 49 Darley Road, BN20 7UR, Eastbourne, UK; 2 Faculty of Health Sciences, University of Southampton, Southampton, Highfield SO17 1BJ, UK

**Keywords:** Questionnaires, Survey, Expectations, Musculoskeletal manipulations, Osteopathic medicine

## Abstract

**Background:**

Patients’ expectations of osteopathic care have been little researched. The aim of this study was to quantify the most important expectations of patients in private UK osteopathic practices, and the extent to which those expectations were met or unmet.

**Methods:**

The study involved development and application of a questionnaire about patients’ expectations of osteopathic care. The questionnaire drew on an extensive review of the literature and the findings of a prior qualitative study involving focus groups exploring the expectations of osteopathic patients. A questionnaire survey of osteopathic patients in the UK was then conducted. Patients were recruited from a random sample of 800 registered osteopaths in private practice across the UK. Patients were asked to complete the questionnaire which asked about 51 aspects of expectation, and post it to the researchers for analysis.

The main outcome measures were the patients-perceived level of expectation as assessed by the percentage of positive responses for each aspect of expectation, and unmet expectation as computed from the proportion responding that their expectation “did not happen”.

**Results:**

1649 sets of patient data were included in the analysis. Thirty five (69%) of the 51 aspects of expectation were prevalent, with listening, respect and information-giving ranking highest. Only 11 expectations were unmet, the most often unmet were to be made aware that there was a complaints procedure, to find it difficult to pay for osteopathic treatment, and perceiving a lack of communication between the osteopath and their GP.

**Conclusions:**

The findings reflected the complexity of providing osteopathic care and meeting patients’ expectations. The results provided a generally positive message about private osteopathic practice. The study identified certain gaps between expectations and delivery of care, which can be used to improve the quality of care. The questionnaire is a resource for future research.

## Background

Osteopathic care in the United Kingdom (UK) comprises an important component of the provision of musculoskeletal services [1,2]. In 2001, there were 4442 statutory-registered osteopaths delivering osteopathic care, based mainly within private practices [3]. While patients’ expectations of osteopathic care have been little researched, there is a considerable body of knowledge from other areas of primary and musculoskeletal care.

Patients’ expectations of their interaction with healthcare are based on cognitive and affective beliefs and values, which evolve in an ‘epi-phenomenal’ way through dynamic interplay between the therapy and therapist, and the patient’s subjective experience of change in symptoms [4-6]. Patients’ expectations are culturally modified and vary with age [7-10], gender [11], ethnicity [12], and social factors such as deprivation and unemployment [13]. They also vary with health condition [14]. Musculoskeletal patients often have no prior expectations of treatment, yet they tend to believe that their symptoms have a physical basis and have views about what type of treatment might be appropriate [15]. The formation of expectations and perceptions of chiropractic care has been found to be rooted in a lay referral system based upon the successful experience of family and friends, and their recommendation [5].

In the context of primary care, the most important expectation are interpersonal care, followed by competence (symptom relief), involvement in decisions, fast access, and information for self-care [16]. Patients with back pain have specific additional expectations of a clear diagnosis of the cause of pain, explanation of the cause of their problem, a physical examination, and confirmation that their pain is real [8,14]. Within complementary therapy, patients also value improved quality of life, avoiding ‘toxic’ drugs and a holistic approach [17-19]. In private musculoskeletal care, patients act as consumers and manage their care, they make choices of therapy and therapist; they expect value for money and “added extras” in the environment [20], and may bench-mark the quality of the service and professional expertise against NHS primary care [21]. Expectations also influence the outcome of (response to) treatment, including satisfaction with treatment [22-24]. It may be beneficial for clinicians to encourage realistic positive expectations through improved explanation of the problem, and shared decision making [25].

The few studies investigating patients’ expectations of osteopathic care have suggested that patients favour the manual nature of manipulation and judge it an appropriate treatment due to its “hands-on” nature [26]. Patients seeking osteopathy or other complementary therapies are likely to have consulted their GP before seeking osteopathic treatment or any form of complementary and alternative medicine (CAM) therapy, and may have received other forms of treatment e.g. physiotherapy, or have tried a selection of CAM therapies before seeking an osteopath [27]; and symptomatic relief was the primary expectation [18]. The desire for an effective and quick resolution to their symptoms was similar whether visiting the osteopath or the GP. Osteopathic studies of patient *satisfaction*[21,28,29], which is related in part to expectations being met, also emphasise the importance of the interpersonal relationship with the osteopath.

The regulator of the osteopathic profession, the General Osteopathic Council (GOsC), commissioned this research as part of a wider programme of work to enhance knowledge of the attitudes, needs and concerns of the public and patients who seek osteopathic care. This occurred within the context of movement towards patient-centred healthcare within UK health policy [30,31]. The study reported here is the final stage of a mixed methods study, the final report to the funder is publicly available [32]. The initial qualitative stage has been reported [33]. The aim of this stage was to quantify the most important expectations of patients consulting osteopaths, and the extent to which those expectations were met or unmet, in private UK osteopathic practices.

## Methods

The methodology chosen to evaluate the expectations of osteopathic patients was a national patient survey, participants being recruited when consulting osteopaths in private practice, and being invited to complete a questionnaire about their expectations of care.

### The questionnaire tool

There was a lack of standardised or validated measurement tools for expectation [14] although a number of measures have been used in specific contexts or for specific conditions [34-37]. There was also inconsistency between studies in the definition of expectation. For this study, a patient-centred definition was used: expectations about aspects of the consultation as perceived and understood by the patient. This definition is similar to that proposed in a recent study in back pain [14].

An osteopathy-specific questionnaire was developed for this study. A formal development process was adopted [38-42]. The question topics were drawn from a literature review [32] and from focus groups and interviews with osteopathic patients [33]. A semi-structured format was adopted for ease of completion by patients, and to permit statistical analysis [38]. Rating scales were used where possible since these produce more information and variance than other types of response [42]; a 5-response scale was selected as being user-friendly and for its statistical properties.

The questionnaire was designed, tested, and piloted in three stages with healthy volunteers and then with osteopathic patients in volunteer practices. More than 70 aspects of expectation were identified initially as candidate questions for the questionnaire. These were refined during the three pilot phases, which involved 45 participants. Fifty-one aspects of expectation were included in the final questionnaire (see Additional file 1), which took about 15 minutes to complete. The final questionnaire comprised four sections:

(1) demographic and personal information;

(2) statements aimed to evaluate which expectations were most prevalent, using Likert rating scales permitting responses from “strongly agree” to “strongly disagree”;

(3) statements to evaluate whether or not the expectation had been met (had “actually happened” or not) when visiting the osteopath;

(4) four open-ended questions enabling patients to articulate any other issues they felt were not covered by the questionnaire.

Factor analysis [43] was undertaken by the study statistician (MH) to determine redundancy and hierarchy of importance of the questions. The factor analysis found that all of the fifty-one questions about expectation contributed information, and none were redundant. The visual appeal and readability of the final version of the questionnaire was improved by use of two font colours (as recommended by http://www.jisctechdis.ac.uk) and professional graphic design services. The questionnaires were identified by a coded study number comprising a study identifier for the osteopath and sequential codes for participants. There were no hidden codes to identify patients, therefore no reminders could be sent to non-responders.

### Recruitment of patients

The strategy for recruitment aimed to obtain a large sample of osteopathic patients, recruited via private osteopathic practices, distributed geographically across the whole of the United Kingdom (UK). To optimise diversity of practice type and location, a large (25%) random sample of osteopaths was used. The sample of osteopaths was drawn with permission from the UK Statutory Register of Osteopaths as published by the General Osteopathic Council in 2009; the sample was stratified to ensure contributions from England, Wales, Scotland, and Northern Ireland. Randomness of sampling from the lists was achieved using integers from a random number generator (http://www.random.org).

The randomly selected osteopaths were contacted to invite them to assist in recruiting patients for the study, and also to ensure that they were in private practice. NHS practices were excluded because NHS ethical approval had not been sought as there were few such practices and their inclusion could have introduced heterogeneity, making the results less robust [44].

Osteopaths were asked to recruit patients for the survey. Patients were eligible if they were currently receiving treatment, had the capacity to give consent, and were able to complete the questionnaire. The exclusion criteria were: not currently receiving osteopathic treatment, unable to understand the questionnaire, or not having the capacity to consent. Children aged less than 16 years were not eligible. The protocol aimed for a systematic sample of patients: that is, all consecutive, eligible patients attending on given days were invited. To maximise patient compliance [45], osteopaths were asked to start recruitment on a Tuesday morning or as soon as possible thereafter. The instructions to osteopaths stressed the importance of strict adherence to protocol to avoid selection bias. Possible deviation from this protocol was evaluated though a short ‘Recruitment form’ which osteopaths were asked to complete and return.

### Sample size

The sample size was calculated based on a need for sufficient statistics to undertake some subgroup analysis, for example by age group or region, for both new and returning patients. The minimum sample size for returned questionnaires was set at 1500 in total including at least 500 new patients, which would provide a 95% confidence interval of 3% or smaller in estimated proportions overall, or 4% for new patients [46].

In similar research using recruitment of patients by practitioners [47] the reported rate of participation by practitioners was as low as 33%. The patient response rate was estimated at 50-70%, leading to an overall anticipated response rate of 16-23%. Therefore, in order to realise the required sample size of 1500 patients, each of the random sample of 800 osteopaths was asked to invite 14 patients to participate, comprising at least 4 consecutive new patients and up to 10 consecutive returning patients.

### Procedure

Each of the 800 selected osteopaths was sent a package of study documentation, containing fifteen Participant Questionnaire Packs for patients. Each pack contained a letter of invitation for the patient, the questionnaire, three versions of the Participant Information Sheets designed for adults of various reading ages (15 years and over; 10–14 years; and 5–9 years, respectively). A reply-paid addressed envelope was included for return of the completed questionnaire to the researchers. Invited patients were given a pack to take home and read at their leisure, and decide whether or not they wished to complete the questionnaire.

### Data entry and analysis

Completed questionnaires were returned to the Clinical Research Centre and the data were input into an EXCEL spread sheet. Accuracy of entry was assured by checking a sample of 5 (10%) of each batch of 50 questionnaires: batches yielding more than 1 error in the sample were returned for re-input of all 50 questionnaires. The maximum error rate was therefore 0.0016 (i.e. 1 in 5 sets of 122 data items), less than 0.2%. The numerical and categorical data from the questionnaires were analysed using the Statistical Package for Social Scientists (SPSS) version 16.0. Data quality and missing values were assessed. The responses to the questions with rating scales were assigned numerical values for analysis.

Two statistics were constructed for each aspect of expectation: (1) the prevalence of positive expectation and (2) the prevalence of unmet positive expectation. Positive expectation was defined as agreement with the statement, and was used as it was intuitively understandable and gave almost identical rankings to the statistically preferable median score. Unmet expectation was a more complex statistic based on the participant’s paired responses from sections D and E of the questionnaire (see Additional file 1) about their expectation and whether or not it they perceived it to have happened. The prevalence of unmet expectation was the percentage of people with positive responses for a given expectation statement who also responded that “it did not happen”. An ‘unacceptable’ level of unmet expectation was defined using concepts from management science of a ‘net promoter score’ [48]. According to this theory, a business is likely to be successful if the percentage of ‘promoters’ of the service minus the percentage of ‘detractors’ is greater than 75%. If patients with unmet expectations are assumed to be detractors, and those whose expectations are met become promoters, the proportion with unmet expectations should not exceed 12.5%.

### Ethical approval

An expert Steering Group was set up by the funder, the General Osteopathic Council, to review project management and progress. Ethics permission was given by the University of Brighton Faculty of Health Research Ethics and Governance Committee; advice was sought also from the regional NHS Research Ethics Committee. Incentives for patients or telephone reminders to osteopaths to encourage them to participate were not permitted by the ethics committee. If the information on a questionnaire were to suggest very serious misconduct by an osteopath, the researchers would have been obliged to trace the practice concerned and to inform the profession’s regulator, the General Osteopathic Council. In the event, no such evidence was found.

## Results

### Data collected

**Figure 1:**
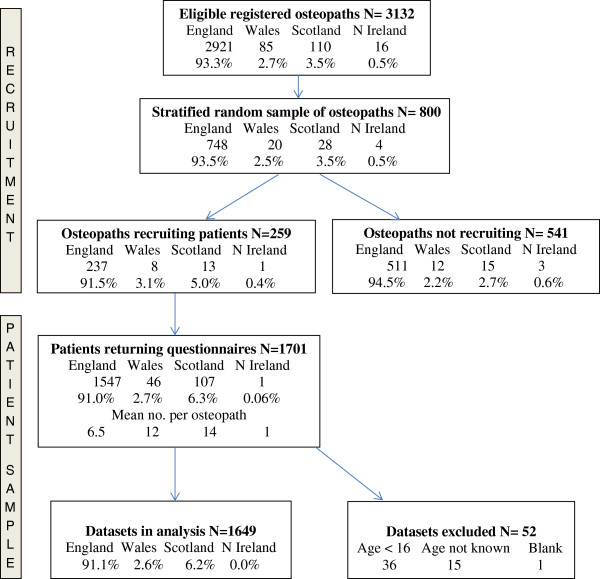
Flow diagram of recruitment, by country.

In total, 11,200 questionnaires were distributed and 1,701 completed questionnaires were returned to the researchers, a response rate of 15.2%. Analysis of the identifier codes on the returned questionnaires showed that the questionnaires came from patients attending 259 (32.4%) of the 800 osteopaths contacted in the study. The number of patient responses per osteopath ranged from 1 to 14 with a mean of 6.3. Formal comparison of responders and non-responders was carried out by country within the UK, the only information publicly available about participants, as shown in Figure 1, the flow diagram of participant recruitment. Participation and recruitment rates per osteopath were slightly higher in Scotland and lower in Northern Ireland than in the majority region, England. No evidence was found of selection bias in recruitment by osteopaths, using the information on the Recruitment forms returned by 151(58%) of participating osteopaths.

### Characteristics of respondents

The characteristics of respondents are shown in Table 1 and are compared to a previous survey [3]. The latter differed in collecting new episodes only, in all age groups, hence the higher proportion of new patients and lower mean age 44.8 (SD +/− 19.1) years compared to 54.0 years (SD +/− 14.9) in this study. The proportion of patients who were new to osteopathy at 17.8% was similar to that in a survey by the General Osteopathic Council in 2001 [49] which reported 17% new patients. However both prior surveys found 40% or more male patients, suggesting that our sample was skewed towards female respondents. Otherwise the characteristics were consistent with prior surveys, with the great majority of patients being white British, and either employed or retired.

**Table 1:** Characteristics of the 1649 respondents included in the analysis

**Characteristic**	**This study**	**This study**	**From Fawkes et al. 2010 [**[3]**]**
	**number**	**%**	**%**
**Gender**			
Male	499	30.3	43.0
Female	1149	69.7	56.0
**Ethnicity**			
White British	1499	90.9	85.1
Other	150	9.1	14.9
**Employment status**			
Employed	655	40.3	45.6
Self employed	295	17.9	16.5
Retired	523	31.7	19.0
Other	166	10.1	18.9
**Naive to osteopathy**			
Yes, new patient	293	17.8	59.0
No, returning patient	1356	82.2	40.0

Data-checking revealed that responders completed the questionnaires thoroughly, with less than 3% missing data for most items. After exclusion of a small number of datasets where the patient’s age was missing or ineligible, 1649 sets of data were included in the analysis.

The general health of respondents was good, only 14.8% considered their general health was fair or poor, and 11.2% considered themselves to have a disability. The majority (58.6%) reported symptoms of moderate severity, though 15.3% considered their symptoms to be severe. For 51.1%, the duration of symptoms was “years” and for 26.9% it was days or weeks. Over 52% had first visited an osteopath 5 or more years ago. Many also had prior experience of physiotherapy (63.1%), or chiropractic (29.8%). More than 90% were self-funding their treatment, with 6.9% funded through insurance and just 0.2% funded by the NHS.

Over 96% of patients agreed or strongly agreed that they were satisfied with their treatment, and only 0.3% (5 patients) were unsatisfied. 4.5% of respondents added free text describing expectations that they considered had not been met.

One of the fifty-one statements in the main section of the questionnaire was about belief rather than expectation: it was worded “I would be prepared to forgo some luxuries to afford osteopathic treatment”. This statement was excluded from the later results which include fifty aspects of expectation. A majority (81.5%) of respondents agreed that they would be prepared to forgo luxuries; only 40.9% perceived that this ‘did not happen’.

### Patients’ most prevalent expectations

‘Prevalent’ was defined as more than 75% of respondents responding positively to a statement. Thirty four (68%) of the 50 aspects of expectation were prevalent, and are shown ranked in Table 2. The corresponding response rates for the 293 (18%) patients who were new to osteopathy were very similar to those for the study population as a whole.

**Table 2:** The 34 most prevalent aspects of expectations of osteopathic care, ranked by % positive responses

**What do you expect when you go to an osteopath?**	**% Positive responses**
I expect to be able to ask questions	99.8%
I expect the osteopath to listen to me	99.1%
I expect to be treated with respect.	98.8%
I expect to be given a clear explanation of my problem that I understand	98.7%
I expect the osteopath to only treat one patient at one time	97.9%
I expect the osteopath to take a detailed account of my clinical history.	97.6%
I expect the clinic environment to be hygienic and professional looking	97.5%
I expect the osteopath to make me feel at ease	97.5%
I expect to be given advice about how to manage my symptoms myself	96.4%
I expect my questions to be answered to my satisfaction	96.2%
I expect to be reassured that the information that I am asked to provide will be kept confidential	96.1%
I expect to be given advice on how to prevent the same problem happening again	94.3%
I expect my osteopathic treatment to be value for money	93.5%
I expect to be given a choice of appointment times	92.8%
I expect the practice to display evidence of the osteopaths professional qualifications	92.7%
I expect the osteopath to monitor my reaction to his/her treatment	92.3%
I expect to be given information about the risks and side effects of treatment	90.7%
I expect to see the same osteopath each time	90.5%
I expect to be able to phone the osteopath for advice if I needed	89.7%
I expect the osteopath to identify my problem area with her/his hands.	89.5%
I expect the osteopath to be sympathetic and caring	88.3%
I expect to be given an explanation of what the treatment will involve before it is given	88.3%
I expect to be given information about the benefits of treatment	86.8%
I expect the consultation to last at least thirty minutes	86.6%
I expect the waiting area to be comfortable and relaxing	84.1%
I expect to be involved in making decisions about my treatment	84.0%
I expect the osteopath to refer me elsewhere if my symptoms are not improving	83.9%
I expect to be given activities or exercises to do at home	80.8%
I expect the practice to make provision for people with disabilities	80.1%
I expect to be asked about effects of previous treatment	80.0%
If I am not satisfied with any part of my treatment I would expect to be given information about how to make a formal complaint	79.6%
I would expect there to be communication between my osteopath and GP if necessary	78.0%
Before my first treatment I expect to be given information about what will happen during treatment.	77.3%
I expect to be given a clear osteopathic diagnosis of my problem at my first appointment.	76.0%

### Unmet expectations

Most (24, 71%) of the 34 prevalent expectations were perceived by respondents to be met well, however for 10 (29%) prevalent expectations the proportion of patients with unmet positive expectations was unacceptable (over 12.5%) or borderline, as shown in Table 3. Five of these prevalent expectations had rather high levels (more than 20%) of unmet positive expectations. In general, new patients had similar though slightly higher levels of unmet expectation than respondents as a whole. However, the percentage of unmet expectations was substantially higher in new patients in relation to three issues, marked by asterisks in Table 3: being asked about the effects of previous treatment, provided with pre-treatment information about what to expect, and being given advice on prevention.

**Table 3:** The ten prevalent expectations that were unmet overall (fourteen unmet for new patients) ranked by % with unmet positive expectations

	**% with unmet expectations**	
**What actually happened during your visits to the osteopath?**	**All respondents**	**New patients only**
I was made aware that there is a complaints procedure should I need to use it	65.63	70.8
There was communication between my osteopath and GP about my problem	33.91	34.68
I was informed of the risks and side effects of the treatment	22.98	23.85
There was access for people with disability	22.46	25.48
The osteopath was able to refer me elsewhere when my symptoms did not improve	21.92	20.75
I was asked about the effects of previous treatment	17.23	36.26*
The osteopath assured me that my details were kept confidential	17.02	21.09
I was given the opportunity to receive advice from the osteopath over the telephone	15.78	15.12
Before my first appointment I was given information about what would happen during treatment.	14.8	25.23*
I was given advice on how to prevent the problem happening again	12.5	20.9*
The osteopath did not treat other patients at the same time as me		13.83
I was given activities and exercises to do at home		15.06
I did see evidence of the osteopaths’ qualifications		14.74
I was given information about the benefits of treatment		12.79

Table 4 provides an overview of the prevalence of expectation (‘customer demand’) tabulated against the level of unmet expectation (’service delivery’). Borderline categories of five percentage points have been created, 2.5% either side of the cut-off levels of 75% for expectation being “prevalent”, and 12.5% for unmet expectation being an acceptable level of customer service, respectively. The table emphasises that 28 of the 50 (56%) of all expectations were met well, and 21 of those 28 (75%) were prevalent expectations. Six expectations (12%) were on the borderline of unacceptable service delivery, three of these being prevalent expectations. Sixteen expectations (32%) were poorly met, of which nine were prevalent expectations. The less prevalent unmet expectations should not be over-looked; they included the use of consent forms and provision of gowns for modesty. Among the patients who *did* expect these items, a high proportion found their expectation unmet.

**Table 4:** Summary of expectations by prevalence (% positive agreement) and the degree to which they were met (% positive expectations that did not happen)*

**Expectations (%)**	**Prevalent expectations (>77.5%)**	**Borderline expectations (72.5-77.5%)**	**Less prevalent expectations (<72.5%)**
**Meeting positive expectations (%)**			
**Expectations met well or adequately (15-100%)**	**21**	**2**	**5**
	*Evidence of qualifications*	*Clear diagnosis*	*Gentle/ vigorous treatment*
	*Information on benefits*	*Privacy for undressing*	*Symptoms improved*
	*Involvement in decisions*		*Pain-free treatment*
	*Self-management advice*		
	*Explanation of treatment and of cause of problem*		
	*Comfortable waiting area*		
	*Choice of appointment time and osteopath*		
	*Value for money*		
	*Case History taken*		
	*Manual examination*		
	*Empathy, respect and listening*		
	*Able to ask questions*		
**Borderline (10–14.9%)**	**3**	**2**	**1**
	*Prevention advice*	***Discomfort after treatment***	*Initial estimate of treatments required*
	***Other patients treated at same time***	*Pre-visit information*	
	*Home exercises advice*		
**Poorly met (<10%)**	**8**	**0**	**8**
	*Informed of complaints procedure; risks; confidentiality*		*Negotiate cost Gown or towel provided*
	*Communication with GP*		*Electrotherapy*
	*Disability access*		*Gender of osteopath*
	*Referral on*		*Able to have chaperone*
	*Asked about prior treatment effects*		*Consent form*
	*Telephone advice*		***Worse after treatment***
			*Initial prognosis*

^*^Bold items in Table 4 denote undesirable rather than desirable aspects of care.

Three of the 50 expectations related to aspects of care that are likely to be undesirable, such as pain and discomfort, or other patients being treated at the same time. For these, unmet expectations could be viewed as beneficial. These undesirable aspects are shown in bold in Table 4.

The nine unmet prevalent expectations were mapped against five broad conceptual themes describing patient expectations which had emerged in the first qualitative phase of the study [33]. There were no unmet expectations that mapped onto the ‘therapeutic process’ or ‘individual agency’ themes. Expecting to be made aware that there was a complaints procedure, disability access and telephone advice all fall within the ‘customer experience’ theme; a perceived lack of communication between their osteopath and their GP, options of referral on, and being asked about the effects of previous treatment fall within ‘professional expertise’; and assurance of confidentiality and information about risks and side effects mapped onto the’ interpersonal relationship’ theme.

## Discussion

The study has provided the first statistically robust profile of the expectations of osteopathic patients, with a reasonable sample size (1,649 in the analysis). Osteopathic patients’ expectations are very complex and encompass at least 50 different aspects of care. More than 75% of the respondents agreed that they did expect 34 (68%) of the 50 expectations statements’. In the ‘top ten’ expectations, there was an emphasis on open exchange of information; and overall they represent an interesting mix of service, conduct, therapeutic relationship, professional expertise, information-giving, and ethical aspects of care.

Most (71%) of the 34 prevalent expectations were met to a good or acceptable level, providing a positive message for the osteopathic profession. Five expectations were particularly poorly met and highlight areas where service quality could be improved. The unmet expectations may reflect the fact that osteopathy as a profession is in the process of moving from the marginalised position of a CAM profession [46] into more mainstream healthcare, for example being recommended within NICE guidelines [2]. Patients are also demanding a higher level of customer service [16]. There were some areas where perhaps more investigation is needed. For example, attitudes to being provided with a towel or gown for modesty, or the need to sign a consent form.

The study results were highly consistent with existing evidence, including a systematic review of patient expectations of treatment for back pain [8], and with previous osteopathic studies [21,26,28].

This study has many implications for practice in osteopathy and possibly in other CAM professions. Osteopaths may need to make certain aspects of their professional decisions more explicit, for example how and when they communicate with the appropriate wider network of health professionals in their area, including the patient’s GP; and how and when they conduct their process of triage at the first appointment, with a view to onward referral if required. Osteopaths can also enhance trust and improve the interpersonal relationship with patients by providing more information about risks and side effects of treatment, and provide reassurance of confidentiality, since most patients expect these. New patients may require more pre-treatment information, be asked to sign a consent form, and offered a towel or gown for modesty.

The main limitation of the study was the possibility of selection bias due to the low response rate. The low participation rate (32%) of the randomly selected osteopaths, despite an active strategy to raise awareness and motivation, was disappointing but not atypical of other studies relying for recruitment from a random sample of practitioners with no incentives [47]. The response rate among patients was higher at around 48% (based on the assumption that only 32% of osteopaths invited patients to participate, so at most 3600 questionnaires were distributed) providing less opportunity for bias. The representativeness of the sample was supported by data from similar previous studies, although participation was slightly skewed by country and gender. Communications from 31 osteopaths who refused to participate included several lengthy letters and anecdotal reasons for non-participation suggested a lack of research awareness and concerns about the value of this research and research in general. The patient respondents were rather homogeneous and appeared typical of the profile of private osteopathic patients nationally [3] in tending to be well educated, white Caucasian, and either employed or retired. Homogeneity increases the robustness of the findings with respect to the research question, but limits their generalisability to populations with different ethnicity or socially less advantaged groups.

The analysis treated all responses as independent data points, but since there were varying numbers from different practices it is possible that the results were skewed by a clustering effect. We did not adjust for this because of the wide range of responses from different practices.

Expectations are not static, they evolve during the course of treatment [50,51] hence the timing of measurements may be important. In this study, post-treatment expectations were collected and appeared to differ surprisingly little between new and returning patients. It would be useful to compare these data with pre-treatment expectations and also to compare expectations in patients with chronic and acute symptoms.

The questionnaire was new and, although un-validated, was thoroughly piloted; it performed well, being completed consistently by respondents with few gaps and no obvious ambiguities in the framing of questions. Only a small number of additional expectations were elicited in the open questions, suggesting that most relevant aspects had been covered. Future minor amendments to be considered include more direct questions about immediate or substantial impact on symptoms, and possible re-framing of the questions on pain and on financial costs. The questionnaire has proved its value and will provide a useful resource for the future.

## Conclusions

This study was the first to directly measure the expectations of osteopathic patients and the extent to which patients’ expectations were met or unmet. In private osteopathic practices, patients’ expectations appeared to be generally met well.

The results provided guidance for patients about what it is reasonable to expect when they seek osteopathic care. The study has identified certain gaps between expectations and the delivery of care, which can be used to improve the quality of care provided by osteopaths, through the regulator via standards, through educators via training, and through the professional body which supports osteopathic practices to improve service delivery.

The questionnaire is now a resource for future research, including surveys in other settings such as the training clinics in osteopathic education institutions, in NHS services or overseas. Further survey research is recommended to confirm the current findings and to evaluate expectations within more diverse populations of osteopathic patients. Slight modification to the questionnaire is recommended to take account of the new aspects identified. Further exploratory research is needed to gain understanding of patients’ expectations about aspects such as communication between the osteopath and the GP, the consent process and arrangements for undressing.

## Abbreviations

CAM: Complementary and alternative medicine; GP: General practitioner; NHS: National Health Service (UK); NICE: National institute for health and clinical excellence (UK).

## Competing interests

The funder of the study was the General Osteopathic Council whose remit as regulator of the profession is to safeguard the interests of patients; their interest was to assure that the study was conducted well and provided accurate information for patients. Three of the authors (Leach, Fawkes, Fiske) were osteopaths in private practice.

## Authors’ contributions

CMJL was principal investigator conducting the study, AM lead the design and testing of the questionnaire and advised on analysis of the survey, MH was the statistician who conducted the analysis, LMB and AF created the participant databases and conducted data quality checking, VC carried out the focus groups and identified the topics for the questionnaire, CAF was the main reviewer of the literature under-pinning the study, APM advised on all stages of the study and analysed the focus group data. All authors read and approved the final manuscript.

## Pre-publication history

The pre-publication history for this paper can be accessed here:

http://www.biomedcentral.com/1472-6882/13/122/prepub

## Supplementary Material

Additional file 1 The study questionnaire. Click here for file
