# Supplementary material for: Patients’ expectations of private osteopathic care in the UK: a national survey of patients
Source: BMC Complement Altern Med. 2013 May 31;13:122. doi: 10.1186/1472-6882-13-122 (PMC3679882; doi:10.1186/1472-6882-13-122)
Supplement: Additional file 1 — The study questionnaire. [file 1472-6882-13-122-S1.pdf]

University of Brighton

Osteopathy – Your Views

This questionnaire is for patients currently receiving osteopathic treatment.

Please read the participant information sheet and decide if you would like to take part in the study.

It should take approximately 15 minutes to complete.

All the information that you provide will be treated with strictest confidence and will not affect your treatment.

Please return the completed questionnaire in the attached stamped addressed envelope.

SECTION A: About You

Please tick **one** answer

1 Are you ☐ Male ☐ Female

2 Age

3 What is your marital status?
☐ Single ☐ Married / partner ☐ Divorced /separated ☐ Widowed

4 How would you describe your ethnicity?

White:
☐ British
☐ Irish
☐ Any other white background, please state

Mixed:
☐ White and Black Caribbean
☐ White and Black African
☐ White and Asian
☐ Any other Mixed background, please state

Asian or Asian British :
☐ Indian
☐ Pakistani
☐ Bangladeshi
☐ Any other Asian background, please state

Black or Black British:
☐ Caribbean
☐ African
☐ Any other Black background, please state

Chinese or other ethnic group
☐ Chinese
☐ Any other, please state

5 How would you describe the area you live in?
☐ Rural area ☐ Urban area

6 At what age did you complete your full-time education?

7 What is your employment status?
☐ Employed ☐ Self-employed ☐ Unemployed ☐ Retired ☐ Other

8 Do you consider yourself to have a disability? ☐ Yes ☐ No
If yes, please give details

SECTION B: Osteopathic Treatment

Please tick **one** box

1 Is this your first visit to an osteopath? ☐ Yes ☐ No

2 If no, approximately how long ago was your first visit?
☐ Less than 1 year ☐ 1-5 years ☐ More than 5 years ☐ More than 10 years

3 Who is paying for this osteopathic treatment?
☐ The NHS ☐ Private health insurance ☐ Yourself

4 Have you ever had any other manual therapy treatments?

Physiotherapy	☐ Yes	☐ No
Chiropractic	☐ Yes	☐ No

Other, please state

SECTION C: Your Symptoms

Please tick **one** box

1 How would you describe your current symptoms?
☐ Mild ☐ Moderate ☐ Severe

2 How long have you suffered with the problem that brought you to your osteopath today?
☐ Days ☐ Weeks ☐ Months ☐ Years

3 How do you rate your general health?
☐ Excellent ☐ Good ☐ Fair ☐ Poor

SECTION D: What do you expect when you go to an osteopath?

Please read each statement and then tick the box that is closest to what you think.

What I expect	Strongly agree	Agree	Neither agree nor disagree	Disagree	Strongly disagree
1 Before my first visit I expect to be given information about what will happen during the consultation	☐	☐	☐	☐	☐
2 I expect to be given an explanation of what the treatment will involve before treatment begins	☐	☐	☐	☐	☐
3 I expect to be given information about the benefits of treatment	☐	☐	☐	☐	☐
4 I expect to be able to negotiate the cost of my treatment sessions if necessary	☐	☐	☐	☐	☐
5 I expect to be given a choice of appointment times	☐	☐	☐	☐	☐
6 I expect to be given information about the risks and side effects of treatment	☐	☐	☐	☐	☐
7 I expect to sign a consent form prior to treatment	☐	☐	☐	☐	☐
8 I expect the practice to display evidence of the osteopath's professional qualifications	☐	☐	☐	☐	☐
9 I expect to have the choice of a male or female osteopath	☐	☐	☐	☐	☐
10 I expect to see the same osteopath each time	☐	☐	☐	☐	☐
11 I expect to be offered a chaperone or permitted to bring my own if I wish	☐	☐	☐	☐	☐
12 I expect the waiting area to be comfortable and relaxing	☐	☐	☐	☐	☐
13 I expect the clinic environment to be hygienic and professional–looking	☐	☐	☐	☐	☐
14 I expect the consultation to last at least 30 minutes	☐	☐	☐	☐	☐
15 I expect the osteopath to only treat one patient at one time	☐	☐	☐	☐	☐
16 I expect to be reassured that the information I am asked to provide will be kept confidential	☐	☐	☐	☐	☐
17 I expect the osteopath to take a detailed account of my personal case history	☐	☐	☐	☐	☐
18 I expect the osteopath to be sympathetic towards me	☐	☐	☐	☐	☐
19 I expect to be involved in making decisions about my treatment	☐	☐	☐	☐	☐
20 I expect the osteopath to make me feel at ease	☐	☐	☐	☐	☐
21 I expect to be given privacy when undressing and dressing during examination and treatment sessions	☐	☐	☐	☐	☐
22 I expect to be provided with a gown or towel when asked to undress	☐	☐	☐	☐	☐
23 I expect the osteopath to identify my problem area using her/his hands	☐	☐	☐	☐	☐
24 I expect to be given a clear osteopathic diagnosis of my problem at my first appointment	☐	☐	☐	☐	☐

What I expect	Strongly agree	Agree	Neither agree nor disagree	Disagree	Strongly disagree
25 I expect the osteopathy treatment to be vigorous	☐	☐	☐	☐	☐
26 I expect the osteopathy treatment to be gentle	☐	☐	☐	☐	☐
27 I expect to receive electrotherapy e.g. ultrasound	☐	☐	☐	☐	☐
28 I expect the osteopath to monitor my reaction to her/his treatment	☐	☐	☐	☐	☐
29 I expect to be treated with respect	☐	☐	☐	☐	☐
30 I expect the osteopath to listen to me	☐	☐	☐	☐	☐
31 I expect to be given a clear explanation of my problem that I understand	☐	☐	☐	☐	☐
32 I expect to be told, at my first appointment, how many treatments I may need	☐	☐	☐	☐	☐
33 I expect my osteopathic treatment to be value for money	☐	☐	☐	☐	☐
34 I would forgo some luxuries to be able to afford osteopathic treatment	☐	☐	☐	☐	☐
35 I expect treatment to be painless	☐	☐	☐	☐	☐
36 I expect my symptoms may get worse temporarily following treatment	☐	☐	☐	☐	☐
37 I expect to be able to ask questions	☐	☐	☐	☐	☐
38 I expect my questions to be answered to my satisfaction	☐	☐	☐	☐	☐
39 I expect to be asked about the effects of previous osteopathic treatment	☐	☐	☐	☐	☐
40 I expect there to be communication between my osteopath and GP if necessary	☐	☐	☐	☐	☐
41 I expect the osteopath to refer me elsewhere if my symptoms are not improving	☐	☐	☐	☐	☐
42 I expect to be given advice about how to manage my symptoms myself	☐	☐	☐	☐	☐
43 I expect to be able to telephone the osteopath for advice if needed	☐	☐	☐	☐	☐
44 I expect to be given advice on how to prevent the same problem happening again	☐	☐	☐	☐	☐
45 I expect to be given activities or exercises to do at home	☐	☐	☐	☐	☐
46 I expect to be given a timeframe for improvement of symptoms	☐	☐	☐	☐	☐
47 I expect my symptoms to improve within the given timeframe	☐	☐	☐	☐	☐
48 I expect to feel some pain or discomfort following treatment	☐	☐	☐	☐	☐
49 I expect to be able to return to my normal activities soon after treatment	☐	☐	☐	☐	☐
50 If I am not satisfied with any part of my treatment I would expect to be given information about how to make a formal complaint	☐	☐	☐	☐	☐
51 I expect the practice to make provision for people with disabilities	☐	☐	☐	☐	☐

SECTION E: We are also interested in what actually happened during your visits to the osteopath

What happened during your visit	Did happen	Did happen to some extent	Did not happen	Not applic -able
1 Before my first visit I was given information about what would happen during the consultation	☐	☐	☐	
2 I was given an explanation of what the treatment involved before treatment began	☐	☐	☐	
3 I was given information about the benefits of treatment	☐	☐	☐	☐
4 I was able to negotiate the cost of my treatment sessions	☐	☐	☐	☐
5 I was given a choice of appointment time	☐	☐	☐	
6 I was informed of the risks and side effects of the treatment	☐	☐	☐	
7 I signed a consent form prior to treatment being given	☐	☐	☐	
8 I saw evidence of the osteopath's qualifications	☐	☐	☐	
9 I was given the choice of a male or female osteopath	☐	☐	☐	
10 I saw the same osteopath on each occasion	☐	☐	☐	☐
11 I was offered a chaperone/brought my own chaperone	☐	☐	☐	☐
12 The waiting area was comfortable and relaxing	☐	☐	☐	
13 The environment was hygienic and professional–looking	☐	☐	☐	
14 The consultation lasted at least 30 minutes	☐	☐	☐	
15 The osteopath did not treat other patients at the same time	☐	☐	☐	
16 The osteopath assured me that my details were kept confidential	☐	☐	☐	
17 The osteopath took my personal case history	☐	☐	☐	
18 The osteopath was sympathetic towards me	☐	☐	☐	
19 I was given the opportunity to be involved in making decisions about my treatment	☐	☐	☐	
20 The osteopath made me feel at ease	☐	☐	☐	
21 I was given privacy when undressing and dressing during examination and treatment sessions	☐	☐	☐	
22 I was provided with a gown or towel when asked to undress	☐	☐	☐	
23 The osteopath examined my specific problem area using her/his hands	☐	☐	☐	
24 I was given a clear diagnosis of my problem at my first appointment	☐	☐	☐	
25 I received vigorous treatment	☐	☐	☐	
26 I received gentle treatment	☐	☐	☐	

What happened during your visit	Did happen	Did happen to some extent	Did not happen	Not Applic-able
27 I received electrotherapy treatment e.g. ultrasound	☐	☐	☐	
28 The osteopath monitored my reactions to her/his treatment	☐	☐	☐	
29 I was treated with respect	☐	☐	☐	
30 The osteopath listened to me	☐	☐	☐	
31 I was given an explanation of the cause of my problem that I was able to understand	☐	☐	☐	
32 I was told, at my first appointment, how many treatments I may need	☐	☐	☐	☐
33 My osteopathic treatment was value for money	☐	☐	☐	
34 I had to forgo some luxuries in order to have osteopathic treatment	☐	☐	☐	
35 The treatment was painless	☐	☐	☐	
36 My symptoms got worse temporarily following treatment	☐	☐	☐	
37 I was able to ask questions	☐	☐	☐	
38 My questions were answered to my satisfaction	☐	☐	☐	
39 I was asked about the effects of previous osteopathic treatment	☐	☐	☐	
40 There was communication between my osteopath and GP about my problem	☐	☐	☐	☐
41 The osteopath referred me elsewhere when my symptoms did not improve	☐	☐	☐	☐
42 I was given advice about how to manage the symptoms myself	☐	☐	☐	
43 I was given the opportunity to receive advice from the osteopath over the telephone	☐	☐	☐	☐
44 I was given advice on how to prevent the problem happening again	☐	☐	☐	
45 I was given activities and exercises to do at home	☐	☐	☐	
46 I was given a timeframe for improvement of my symptoms	☐	☐	☐	
47 My symptoms did improve within the given timeframe	☐	☐	☐	☐
48 I felt some pain or discomfort following treatment	☐	☐	☐	
49 I was able to return to my normal activities soon after treatment	☐	☐	☐	
50 I was made aware that there is a complaints procedure should I need to use it	☐	☐	☐	
51 There was access for people with disabilities	☐	☐	☐	

SECTION F: Your experience of osteopathy – what matters to you

1 Please record your three most important expectations of your osteopathic care

1 ______________________________

2 ______________________________

3 ______________________________

2 I am satisfied with my treatment

☐ Strongly agree ☐ Agree ☐ Neither agree/disagree ☐ Disagree ☐ Strongly disagree

3 Do you have any other expectations of osteopathic care that have not been met?

☐ Yes ☐ No

If yes, please give details

____

____

____

4 Did you receive any other type of treatment you were not expecting? Please give details

____

____

____

5 Did you experience anything else during your visit that you were not expecting?

Please give details

____

____

____

Thank you for taking the time to complete this questionnaire

Please return the questionnaire using the envelope supplied:
OPEn project
Clinical Research Centre for Health Professions
Aldro Building
49 Darley Rd
Eastbourne
BN20 7UR

University of Brighton
